# Supplementary material for: Identification and functional modelling of plausibly causative cis-regulatory variants in a highly-selected cohort with X-linked intellectual disability
Source: PLoS One. 2021 Aug 13;16(8):e0256181. doi: 10.1371/journal.pone.0256181 (PMC8362966; doi:10.1371/journal.pone.0256181)
Supplement: S1 File — (DOCX) [file pone.0256181.s002.docx]

| **Table of Contents** | **Page Number** |
| --- | --- |
| S1 Fig: Summary of Variant Filtering Pipeline Decision Tree | 2 |
| S2 Table: Summary of segregation analysis | 3 |
| S3-33 :Pedigrees of Families Used in Segregation Analysis | 4-34 |
| S34 Fig:TENM1 dual-color reporter transgenic lines | 35 |
| S35 Fig:FMR1 dual-color reporter transgenic lines | 36 |
| S36 Fig:POLA1/PCYT1B dual-color reporter transgenic lines | 37 |
| S37 Fig:ARHGEF6 dual-color reporter transgenic lines | 38 |
| S38 Fig:KDM6A dual-color reporter transgenic lines | 39 |
| S39 Fig:AFF2 dual-color reporter transgenic lines | 40 |
| S40 Fig: Western blot to confirm morpholino mediated Six3 knockdown | 41 |
| S41 Fig:*Fmr1* Whole-mount In Situ Hybridization in Mouse Embryos | 42 |
| S42 Fig: Quantification of *Fmr1* transcripts using quantitative PCR | 43 |
| S43 Fig:*Fmr1* quantification using RNA Sequencing | 44 |
| S44 Fig: Evolutionary Conservation of the 6 CRE Surviving Filtering | 45 |
| S3 Table: List of primers used in the study. | 46 |
| S1 Note : Details of Guide RNA and Repair Template Sequences | 47 |
| S2 Note: Metabolic labeling and Basal Protein Synthesis | 48 |
| S3 Note: Hippocampal slice electrophysiology | 49 |
| S4 Note: RNAscope assay | 50 |
| S5 Note: RNA Sequencing | 51 |
| S6 Note: Western blotting | 51 |
| References | 51 |

# **S1 Fig: Summary of Variant Filtering Pipeline Decision Tree**

###
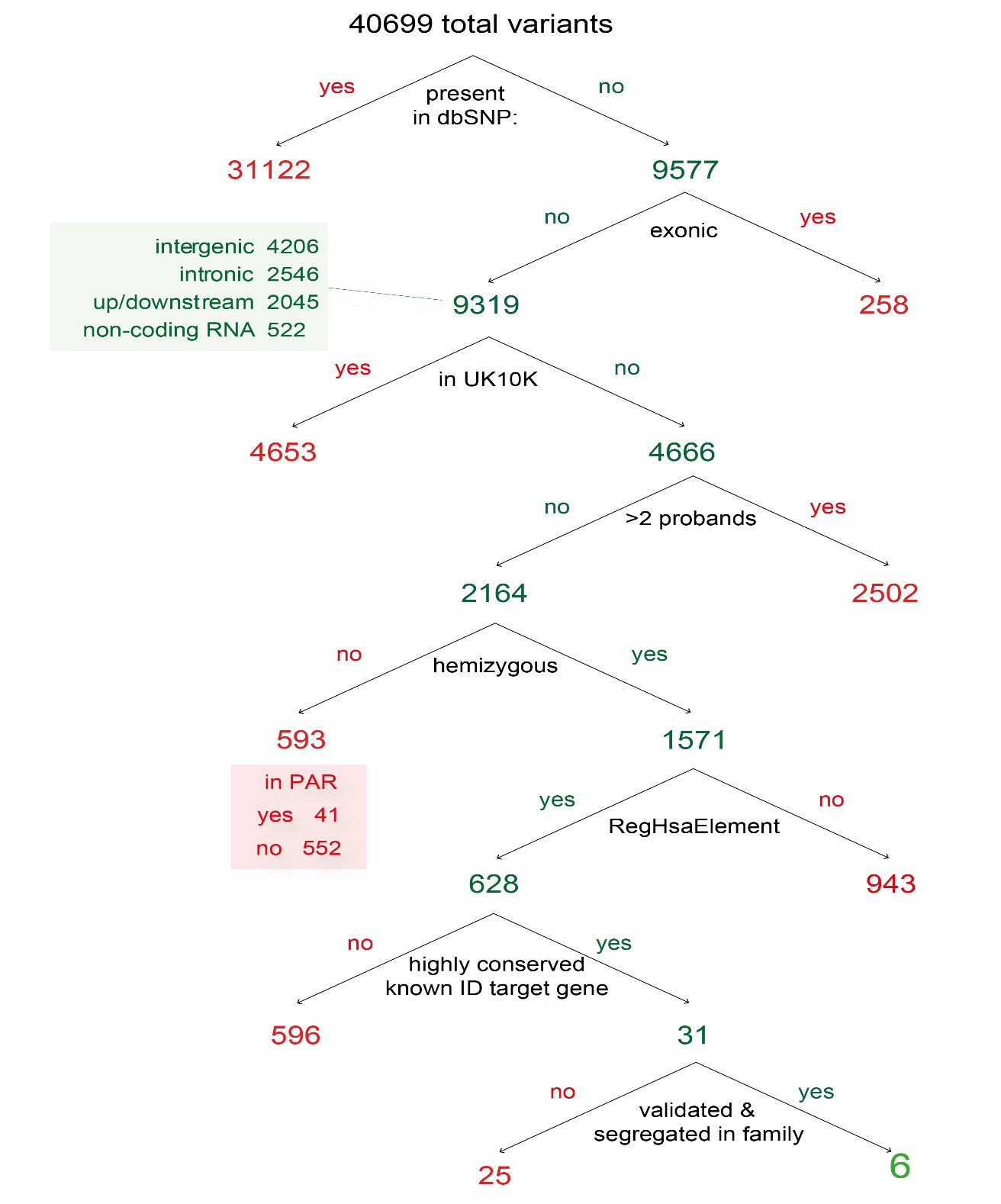


**S2 Table: Summary of segregation analysis**

| **Chr** | **position**  **(hg19)** | **Ref** | **Alt** | **Gene/genes within 1.5Mb region** | **Sample ID**  **1** | **Sample ID**  **2** | **Validation variant Sanger**  **sequencing in the index** | **Tracking in additional affected family**  **members** | **Tracking in unaffected family**  **members** | **Summary** | **Frequency hemizygote individuals**  **in gnomAD** |
| --- | --- | --- | --- | --- | --- | --- | --- | --- | --- | --- | --- |
| X | 147866225 | C | A | *AFF2* | S31 | 391-01 | variant confirmed | DNA not available | DNA not available | DNA not available for tracking | 2 out of 21750 alleles in total |
| X | 99625573 | T | A | *TSPAN6* | S37 | 061-05 | variant confirmed | 61-11 (wt); 61-23 (mut) |  | Did not segregate as expected | 0 |
| X | 136354272 | G | A | *RBMX* | S19 | 136-01 | variant confirmed | DNA not available | 136-06 (wt); 136-08 (mut) | Did not segregate as expected | 0 |
| X | 135962942 | G | C | *ARHGEF6 BRS3* | S37 | 061-05 | variant confirmed | 61-07; 61-11; 61-19; 61-20; 61-23 (all mut) | 061-17 (wt); 061-01 (mut) | Did not segregate as expected | 0 |
| X | 24819218 | G | C | *PDK3 PCYT1B POLA1 EIF2S3* | S45 | 375-01 | variant confirmed | 375-02 (wt); 375-04 (wt) |  | Did not segregate as expected | 0 |
| X | 148000915 | A | G | *AFF2* | S33 | 102-01 | variant confirmed | 102-07 (wt) |  | Did not segregate as expected | 0 |
| X | 136183176 | G | A | *ARHGEF6* | S24 | 409-01 | variant confirmed | 409-02 (wt/mut); 409-03 (wt/mut) |  | Segregated as expected | 1 out of 21716 alleles in total |
| X | 123707760 | T | C | *XIAP* | S27 | 401-01 | variant confirmed | 401-02 (wt); 401-03 (mut) |  | Did not segregate as expected | 0 |
| X | 139187167 | A | G | *MCF2 ATP11C SOX3* | S38 | 006-09 | variant confirmed | 006-16 (wt) |  | Did not segregate as expected | 0 |
| X | 39701238 | C | G | *BCOR* | S18 | 083-01 | variant confirmed | 083-02 (wt); 083-03 (wt) |  | Did not segregate as expected | 2 out of 21657 alleles in total |
| X | 40406651 | T | C | *MED14 ATP6AP2 BCOR* | S45 | 375-01 | variant confirmed | 375-02 (wt); 375-04 (wt) |  | Did not segregate as expected | 0 |
| X | 25260740 | A | G | *POLA1 PCYT1B* | S19 | 136-01 | variant confirmed | DNA not available | 136-06 (wt); 136-08 (wt) | Segregated as expected | 0 |
| X | 106176958 | A | C | *TBC1D8B* | S35 | 029-01 | variant not confirmed |  |  | The variant was not confirmed | 2 out of 21701 alleles in total |
| X | 85932752 | A | G | *CHM* | S18 | 083-01 | variant confirmed | 083-02 (wt); 083-03 (wt) |  | Did not segregate as expected | 0 |
| X | 33131770 | G | C | *DMD* | S45 | 375-01 | variant confirmed | 375-02 (wt); 375-04 (mut) |  | Did not segregate as expected | 0 |
| X | 124269322 | G | A | *ODZ1* | S24 | 409-01 | variant confirmed | 409-02 (wt/mut); 409-03 (wt/mut) |  | Segregated as expected | 0 |
| X | 15010623 | G | A | *PIGA GLRA2 MOSPD2 ASB11 ASB9* | S38 | 006-09 | variant confirmed | 006-16 (wt) |  | Did not segregate as expected | 0 |
| X | 117482305 | T | C | *WDR44* | S44 | 351-01 | variant confirmed | 351-02 (wt); 351-03 (mut) |  | Did not segregate as expected | 0 |
| X | 104365258 | A | G | *IL1RAPL2* | S31 | 391-01 | variant confirmed | DNA not available | DNA not available | DNA not available for tracking | 0 |
| X | 116741775 | C | T | *KLHL13* | S11 | 010-01 | variant confirmed | 010-02 (wt); 010-04 (mut) |  | Did not segregate as expected | 0 |
| X | 146875009 | C | T | *FMR1* | S3 | 347-01 | variant confirmed | 347-03 (mut); 347-04 (mut) |  | Segregated as expected | 0 |
| X | 103994885 | G | A | *FAM199X* | S6 | 440-01 | variant confirmed | DNA not available | DNA not available | DNA not available for tracking | 0 |
| X | 133105579 | G | A | *HPRT1 FAM122B GPC3 FAM122C* | S4 | 370-03 | variant confirmed | 370-01 (wt) |  | Did not segregate as expected | 2 out of 21414 alleles in total |
| X | 45375111 | C | G | *KDM6A* | S43 | 383-01 | variant confirmed | var 383-02 (mut) |  | Segregated as expected | 0 |
| X | 135112643 | T | G | *SLC9A6* | S30 | 382-02 | variant confirmed | 382-04 (wt); 382-05 (wt) |  | Did not segregate as expected | 0 |
| X | 39719711 | T | C | *BCOR* | S7 | 1319-01 | variant confirmed | DNA not available | DNA not available | DNA not available for tracking | 0 |
| X | 38741210 | T | A | *OTC SRPX SPAN7-RP5-972B16.2.1,* and othe | S40 | 439-01 | variant confirmed | DNA not available | DNA not available | DNA not available for tracking | 0 |
| X | 39976350 | C | G | *MED14 ATP6AP2 BCOR* | S22 | 429-01 | variant confirmed | 429-06 (wt); 429-08 (wt) |  | Did not segregate as expected | 0 |
| X | 128915530 | C | G | *AIFM1 OCRL RAB33A ELF4,* and others | S16 | 438-01 | variant confirmed | DNA not available | DNA not available | DNA not available for tracking | 0 |
| X | 39377898 | A | G | *BCOR* | S18 | 083-01 | variant confirmed | 083-02 (wt); 083-03 (wt) |  | Did not segregate as expected | 1 out of 21699 alleles in total |
| X | 34895739 | T | C | *TMEM47* | S45 | 375-01 | variant confirmed | 375-02 (mut); 375-04 (wt) |  | Did not segregate as expected | 0 |

**Chr = chromosome; Ref = Reference allele; Alt = Alternative allele; wt= wild type; mut = mutation**

## chrX:g.147866225C>A the variant was confirmed in the studied individual; DNA from other family members was not available for segregation


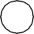


391-01

mut

chrX:g.99625573T>A, the variant did not segregate as expected in the family


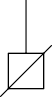

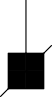

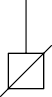

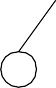

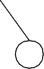


061-09 061-01

061-08 061-18

061-14

061-24 061-04 061-15 061-03

061-16 061-11 061-19 061-20 061-23

wt mut

061-05 061-17

mut

061-22 061-21

061-06

061-10

061-12 061-02

061-07

mut

136- 06


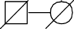

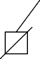

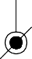

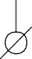

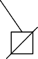

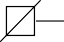

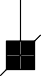

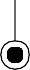

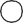

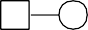

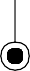

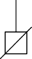

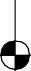


136- 07

136- 02

136- 03 136- 04

136- 09

136- 08

136- 10

136- 01

mut

136- 05

wt


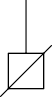

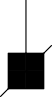

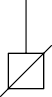

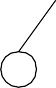

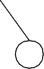


061-09 061-01

mut

061-08 061-18

061-14

061-24 061-04 061-15 061-03

061-16 061-11

mut

061-19

mut

061-20 061-23 061-05 061-17 061-22 061-21

061-06

mut

mut

mut wt

061-10

061-12 061-02

061-07

mut


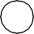


375-03

375-04

wt

375-01 375-02

mut wt


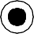

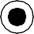

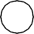

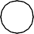

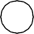

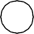

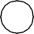

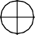

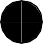

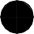

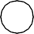

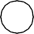

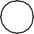

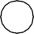

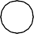


102-15

102-13 102-07

wt

102-12

102-14

102-11

102-01

mut

409-01 409-02


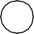

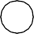

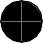

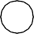

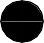

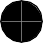

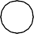


409-03

wt/mut

mut wt/mut

401-02 401-01

24/8/90 23/3/93 401-03

28/3/96

ADHD cerebral brain

palsy damage

following accident

**FAMILY 401**

401-04 401-06

29/12/69 20/7/73

401-05

14/12/39


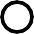

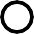

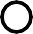

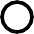


401-01

mut

401-02 401-03

wt

mut


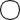

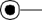

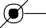

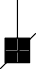

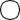

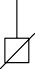

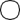

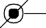

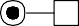

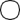

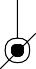

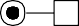

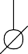


006-05

006-02 006-17

006-01

006-23

006-09

mut

006-14

006-19 006-20

006-16 006-18

wt


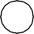


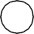


|  |  |  |
| --- | --- | --- |

083-02 083-01 083-03 083-04


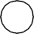

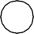

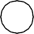

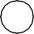

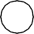

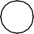

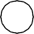

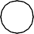

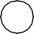

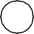

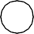

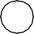


083-08

083-09 083-07

083-10

wt mut wt


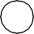


375-03

375-04

wt

375-01 375-02

mut wt

wt

136- 06


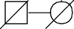

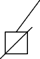

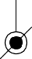

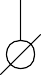

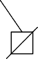

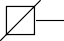

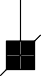

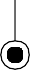

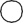

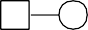

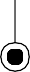

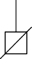

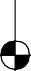


136- 07

136- 02

136- 03 136- 04

136- 09

136- 08

136- 10

136- 01

mut

136- 05

wt


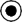

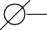

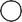

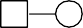

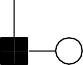

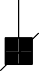

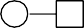


029-03

029-02

029-09

029-08 029-07

029-04

029-05

029-01

wt

029-11

029-06

|  |  |  |
| --- | --- | --- |

083-02 083-01 083-03

083-08

083-09 083-07

083-10

wt mut wt

083-04

375-03

375-04

mut

375-01 375-02

mut wt

409-01 409-02

409-03

wt/mut

mut wt/mut

006-05

006-02 006-17

006-01

006-23

006-09

mut

006-14

006-19 006-20

006-16 006-18

wt

351-01 351-02 351-03

mut wt mut

# was not available for segregation

391-01

mut

010-06 010-02 010-05

wt

010-03

010-01

mut

010-04

mut

mut

mut mut

# was not available for segregation

440-01

mut

mut

wt

383-03

383-01 383-02

mut mut

382-05

wt

382-04

wt

382-03 382-02 382-01

mut

391-01

1319-01

mut

439-01

mut

429-08

wt

429-06

wt

429-01

mut

# was not available for segregation

438-01

mut

|  |  |  |
| --- | --- | --- |

083-02 083-01 083-03 083-04

083-08

083-09 083-07

083-10

wt mut wt

Family 083, chrX:g.39377898A>G, the variant did not segregate as expected in the family

375-03

375-04

wt

375-01 375-02

mut mut

**S34 Fig: *TENM1* dual-color reporter transgenic lines**

**S35 Fig: *FMR1* dual-color reporter transgenic lines**

**S36 Fig: *POLA1/PCYT1B* dual-color reporter transgenic lines**

**S37 Fig: *ARHGEF6* dual-color reporter transgenic lines**

**S38 Fig: *KDM6A* dual-color reporter transgenic lines**

**S39 Fig: *AFF2* dual-color reporter transgenic lines**

**S40 Fig: Western blot to confirm morpholino mediated Six3 knockdown**

The efficiency of Six3 depletion in zebrafish embryos injected with Six3 morpholinos (Six3 Mo) is demonstrated by Western blotting of protein extract from pooled morpholino-injected embryos(after 48 and 72 hrs post injection) with anti-Six3 antibody.Six3 was absent in the extract from Six3 morpholino-injected embryos while Six3 is present in embryos injected with a control morpholino (Ct Mo). Antibody against β-Actin was used as loading control.

**S41 Fig: *Fmr1* Whole-mount In Situ Hybridization in Mouse Embryos**

Whole-mount in situ hybridization for *Fmr1* shows loss of expression of *Fmr1* in the nasal placode in *Fmr1^CRE^* mutant embryos (b) as compared to wild-type embryos (a). Embryos at 13.5 dpc were genotyped (using DNA obtained from one limb for each embryo) and sex matched. They were subsequently probed for *Fmr1* expression. Fmr1*^CRE^* mutant embryos were allowed an enhanced exposure time of 30 minutes in the chromogenic substrate to ensure absence of signal observed is not due to insufficient hybridization.

**S42 Fig:** **Quantification of *Fmr1* transcripts using quantitative PCR**

Transcripts level of *Fmr1* is quantified and normalized with *Gapdh* transcripts levels in the forebrain, midbrain and hindbrain of *Fmr1*^CRE^ knockin mutant as compared to wild type littermates at different stage of development (P7 and P14).Two different exonic regions were used to compare the *Fmr1* transcripts levels. The levels of *Fmr1* transcripts were not significantly different between *Fmr1*^CRE^ knock-in mutant as compared to wild type littermates in different tissues as well as postnatal developing stages (P-7 and P-14).N represent number of biological replicates used and three technical replicates were analyses for each sample. Data are shown as the mean of relative fold change gene expression ± SE. To compare the statistic difference between any pair of data t-test was used to calculate the p value and was considered significant when it is less than 0.05.

**S43 Fig:** ***Fmr1* Quantification using RNA Sequencing**

DESeq2 was used to quantify the Log2 normalised *Fmr1* transcripts levels in the forebrain, midbrain and hindbrain of 3 biological replicates of *Fmr1*^CRE^ knockin mutant as compared to wild type littermates The Log2 normalised *Fmr1* transcripts counts were not significantly different between *Fmr1*^CRE^ knockin mutant as compared to wild type littermates in forebrain, midbrain and hindbrain at P-25 stage of development.

**S44 Fig: Evolutionary Conservation of the 6 CRE Surviving Filtering**

Conservation across 9 species for the 100 bases surrounding the mutated base in the CRE shown in Fig 1B. These figures were adapted from the output of UCSC Genome Browser

**Table S3: List of primers used in the study**

| **Primer details for making transgenic zebrafish** | |
| --- | --- |
| ***TENM1* CRE** **F** | AAGAGAAAAGTGGATGGGAGAGA |
| ***TENM1*CRE** **R** | TCATCTTTATTGAGAAGAATATGGGAC |
| ***FMR1* CRE** **F** | ATTAAGATTCAAGTTCGTTATAGCACT |
| ***FMR1*CRE** **R** | GAAAGTGATATGACAATGGCAACT |
| **Genotyping primers for transgenic mice** | |
| ***TENM1* CRE** **F** | GCTCTCAAATATTGTGTGACAAAG |
| ***TENM1*CRE** **R** | ACCATCACAGGTAGACCTCCTA |
| ***FMR1* CRE** **F** | GAAGAAGCACTCTCCTAAAATGG |
| ***FMR1*CRE** **R** | GCCTTCTCCCTGGTTTATCC |
| **qRT-PCR primers** | |
| ***Fmr1* 6 F (exon14-15)** | CGCGGTCCTGGATATACTTC |
| ***Fmr1* 6 R (exon14-15)** | CTCTGCGCAGGAAGCTCT |
| ***Fmr1* 77 F (exon5-6)** | GATTCCATTCCATGATGTGAGA |
| ***Fmr1* 77 R (exon5-6)** | GCTCTTTTTCATTTGCTCTGG |
| ***Gapdh* F** | GGGTTCCTATAAATACGGACTGC |
| ***Gapdh* R** | CCATTTTGTCTACGGGACGA |
| ***In situ* probe primer sequence for zebrafish and mouse** | |
| ***zfodz1* F** | TTTAGAGTGGCCCACAGACC |
| ***zfodz1* R** | GGAGACTTCAGCTTGGCATC |
| ***zfFmr1* probe** | Gift from Steve Wilson Lab |
| ***mFmr1* F** | GCAGCTTGCCTCAAGATTTC |
| ***mFmr1* R** | AGCCTTGGGTTCAGGTTTCT |
| ***mOdz1* F** | CATCACCTGACCATGCACTC |
| ***mOdz1* R** | TGGGTGGTGGTGAGTAGACA |

**S1 Note: Details of Guide RNA and Repair Template Sequences**

**gRNA *Tenm1*^CRE^***:* AATATTATTAGCCACACATT **TGG**

**Repair template *Tenm1*^CRE^:** AAAGATTGAAATAATTTTATAGAAAGCTTAAAAGTTAAATTCTTCATTATAAATCTCATTGGTTATATGTAGAGTTTCAGATAAACAGAATGACTGTCGAATGATATTTACCAAATGTGTGGCTAATAATATTCCCCCACTTCTAAATATGCATCACTTTTATTGACATAAAAAATATAT

**gRNA *Fmr1*^CRE^***:* ACCTTGTGTCTATGACTATT **TGG**

**Repair template *Fmr1*^CRE^:** TAAGATGGATTCATATTAGGGCTCAAATGCATTGATAGCATTCTACATATTTTTATCCATTTTTATTCCAAGCTACTTTTATCCAAATAGTTATAGACACAAGGTTATTGCAAATTGTATTTGTCTGCTGCCATAGTGCTTTCTATTTTAGAGGAGTAGAAGTAACTATCTCCTTAACAA

Guide RNA and Repair template sequence used to create human variant in *Tenm1* and *Fmr1* CRE in mouse. PAM sequence in the guide RNA is marked in bold and variant nucleotide in repair template is highlighted.

##### **S2 Note: Metabolic labeling and Basal Protein Synthesis**

Juvenile (P25–P32) male littermate WT and *Fmr1*^CRE^ knock-in mutant mice were anesthetized with isofluorane, and the hippocampus dissected into ice-cold artificial cerebral spinal fluid (ACSF) (in mM: NaCl: 124, KCl: 3, NaH2PO4: 1.25, NaHCO3: 26, dextrose: 10, MgCl2: 1, CaCl2: 2, saturated with 95% O2 and 5% CO2). Slices (500 µm thick) were prepared using a Stoelting Tissue Slicer, and within 5 min transferred into 32.5°C ACSF (saturated with 95% O2 and 5% CO2) for 3.5–4 h to allow for recovery of protein synthesis (Sajikumar et al., 2005)^1^. 25 µM ActD was then added for 30 min to inhibit transcription. Slices were incubated in 10 µCi/ml 35S-Met/Cys (express protein labelling mix, Perkin Elmer) for another 30 min to measure protein synthesis. Slices were homogenized in ice-cold homogenization buffer (10 mM HEPES pH 7.4, 2 mM EDTA, 2 mM EGTA, 1% Triton X-100, protease inhibitors tablets (Roche), and phosphatase inhibitors (cocktails 1+2,Sigma Aldrich), and precipitated using trichloroacetic acid (TCA; 10% final) for 10 min on ice and pelleted by spinning at 21,000×g for 10 min. The pellet was washed with ice-cold ddH2O and resuspended in 1 N NaOH then adjusted to a neutral pH with HCl. Triplicate aliquots were added to the scintillation cocktail (Optiphase, Perkin Elmer) and read with a scintillation counter. Averaged triplicate counts per minute (CPM) per µg protein were calculated. To control for daily variation in incorporation rate, the values obtained on each day were normalized to the 35S-Met/Cys ACSF used for incubation, and the average incorporation of all slices analyzed in that experiment, as described (Lipton and Raley-Susman, 1999)^2^.

**S3 Note: Hippocampal slice electrophysiology**

Horizontal hippocampal slices (400 μm) were prepared from postnatal day 25–32 (P25–32) male WT and *Fmr1*^CRE^ knock-in mutant mice. Slices were collected in carbogenated (95% oxygen, 5% CO2) ice-cold dissection buffer containing the following (in mM): 86 NaCl, 1.2 NaH2PO4, 25 KCl, 25 NaHCO3, 20 glucose, 75 sucrose, 0.5 CaCl2, and 7 MgCl2. Slices were incubated for 30 min at ∼30°C in artificial CSF (ACSF) containing the following (in mM): 124 NaCl, 1.2 NaH2PO4, 25 KCl, 25 NaHCO3, 20 glucose, 2 CaCl2, and 1 MgCl2, bubbled with 95% oxygen and 5% CO2. An incision was made through CA1–CA3 boundary, and slices were left to recover for a minimum of 1 h at room temperature (20−22°C) before any recordings were made. For electrophysiological recordings, slices were placed in a submersion chamber heated to 30°C and perfused with carbogenated ACSF at a rate of 4 ml/min.

Field EPSPs (fEPSPs) were recorded at Schaffer collateral/commissural inputs to CA1 pyramidal neurons using extracellular recording electrodes (1–3 MΩ) filled with ACSF and placed in the stratum radiatum layer of the CA1 area. Synaptic responses were evoked by applying single current pulses to the Schaffer collateral/commissural axons using a bipolar stimulating electrode. Stimuli (200μs duration, 30s interval) were set to produce 40–60% of the maximal response amplitude. Metabotropic glutamate receptor-dependent LTD (mGluR-LTD) was induced by acute application (5 min) of the group 1 mGluR agonist 3,5-dihydroxyphenylglycine (DHPG; 100 μM).

Electrophysiological traces were collected using WinLTP (University of Bristol, Bristol, UK) and exported to Microsoft Excel. The magnitude of LTD was calculated by dividing the average fEPSP slope from 50 to 60 min after DHPG application by the average fEPSP slope during the 20 min baseline before DHPG application. Statistical analysis was performed using GraphPad Prism (GraphPad Software). Time-matched normalized data were averaged across experiments and expressed as means ± SE. Significant differences between the WT and Fmr1*^CRE^* knock-in mutant mice were determined using Student's t test.

**S4 Note: RNAscope assay**

Whole brain were dissected out from male WT and *Fmr1*^CRE^ knock-in mutant mice(P-25) followed by fixation with 4% paraformaldehyde and processed for cryostat sections (20 μm). The Sagittal sections were subjected to RNAscope Multiplex Fluorescent Assay. The sections were incubated with pretreatment 2 (RNAscope Target Retrieval Reagents, Advanced Cell Diagnostics) for 30 minutes at 40°C in a HybEZ oven (HybEZ Hybridization System, Advanced Cell Diagnostics), followed by pretreatment 3 (RNAscope Protease III, Advanced Cell Diagnostics) containing protease for 30 minutes at 40°C. After pretreatment 3, the sections were washed with 5× deionized water and incubated with the prewarmed mixed mRNA target probes (FMR1- C2, 496399 and PAX-6-C1, 412821 [Advanced Cell Diagnostics]) for 2 hours at 40°C in the HybEZ oven. The sections were processed in wash buffer for 5 minutes and then incubated with Amp1 for 30 minutes at 40°C and washed with washing buffer for 5 minutes. The procedure was repeated for Amp2, Amp3, and Amp4 AltA for C2 (Cy5), and C1 (FITC) module, After washing with washing buffer for 5 minutes, the sections were cover slipped with Vectashield-DAPI mounting medium (Vector Laboratories).

**S5 Note: RNA Sequencing**

Total RNA was extracted from dissected fore brain, mid brain and hind brain of 3 biological replicates P-25 wild type and *Fmr1*^CRE^ knockin mutant mice from same litter using the RNeasy kit (QIAGEN) combined with QIAshredder (QIAGEN), following the manufacturer’s instructions.For RNA-sequencing, random primed cDNA from poly(A) selected RNA was converted into an Illumina sequencing library using RNA Library Prep Kit from Illumina (E7420, NEB, USA) in conjunction with NEBNext® Multiplex Oligos for Illumina (E7335/E7500, NEB, USA). and single-end 50-base pair (bp) reads were generated using a NextSeq 500 (Illumina Inc, SY-415-1002). Eighteen libraries were combined in two equimolar pools of 9 based on the library quantification results and each pool was run across a single High-Output Flow Cell. Sequencing was performed at the Wellcome Trust Clinical Research Facility (WTCRF; Edinburgh).

Fastq files were processed to transcript-level counts and quality control performed using the bcbio_nextgen pipeline and the illumina-RNAseq workflow template. Differential Expression (DE) analysis was performed in R. The R package bcbio-RNAseq was used to import salmon transcript level counts into DESeq2.

**S6 Note: Western blotting**

Nuclear extract was prepared from ~100 morpholino injected embryos at 48 and 72 hpf using NE-PER Nuclear and Cytoplasmic Extraction Reagents (Thermo Scientific, catalogue number 78833). The extracts were boiled with 30 μL of loading buffer (12.5mM Tris at pH 6.8, 2% SDS, 20% glycerol, 0.002% bromphenol blue, 10% 2-mercaptoethanol) for 5min and were resolved by 10% SDS-PAGE, transferred to nitrocellulose, incubated with antibody (anti-Six3 antibody ab139317,Abcam), and detected by chemiluminescence (Thermo scientific SuperSignal West Femto Maximum Sensitivity Substrate).The membrane was probed with anti-β-actin antibody (ab209869,Abcam) as loading control.

**References**

1. Sajikumar S, Navakkode S, Frey JU. Protein synthesis-dependent long-term functional plasticity: methods and techniques. Curr Opin Neurobiol. 2005;15(5):607-13. Epub 2005/09/10. doi: 10.1016/j.conb.2005.08.009. PubMed PMID: 16150586.

2. Lipton P, Raley-Susman KM. Autoradiographic measurements of protein synthesis in hippocampal slices from rats and guinea pigs. Methods. 1999;18(2):127-43. Epub 1999/06/05. doi: 10.1006/meth.1999.0766. PubMed PMID: 10356343.
