# Supplementary material for: Identification and functional modelling of plausibly causative cis-regulatory variants in a highly-selected cohort with X-linked intellectual disability
Source: PLoS One. 2021 Aug 13;16(8):e0256181. doi: 10.1371/journal.pone.0256181 (PMC8362966; doi:10.1371/journal.pone.0256181)
Supplement: S1 Raw images — (PDF) [file pone.0256181.s003.pdf]

Fig 4G

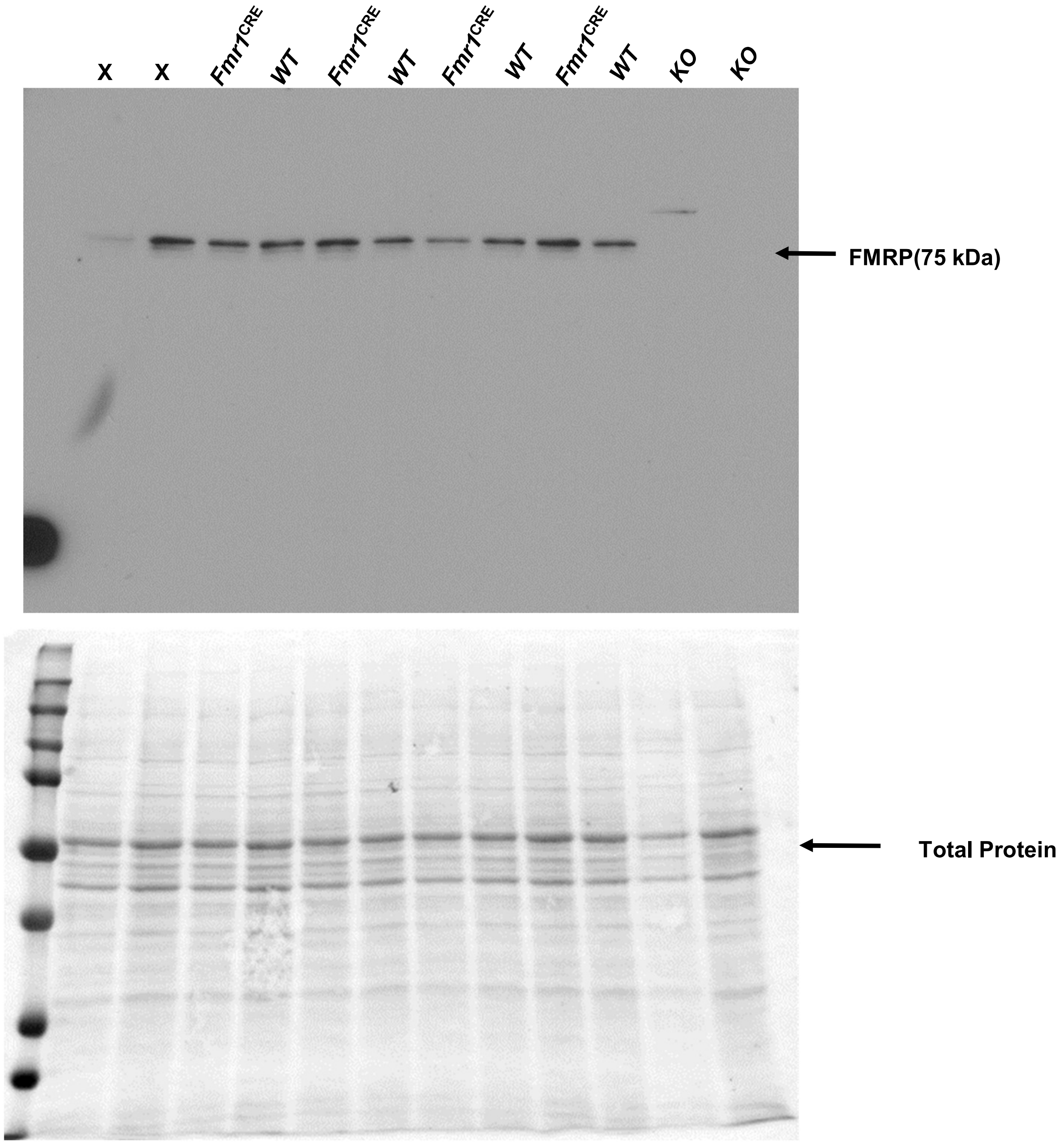
X
X
Fmr1CRE
WT
Fmr1CRE
WT
Fmr1CRE
WT
Fmr1CRE
WT
KO
KO
FMRP(75 kDa)
Total Protein

S40 Fig

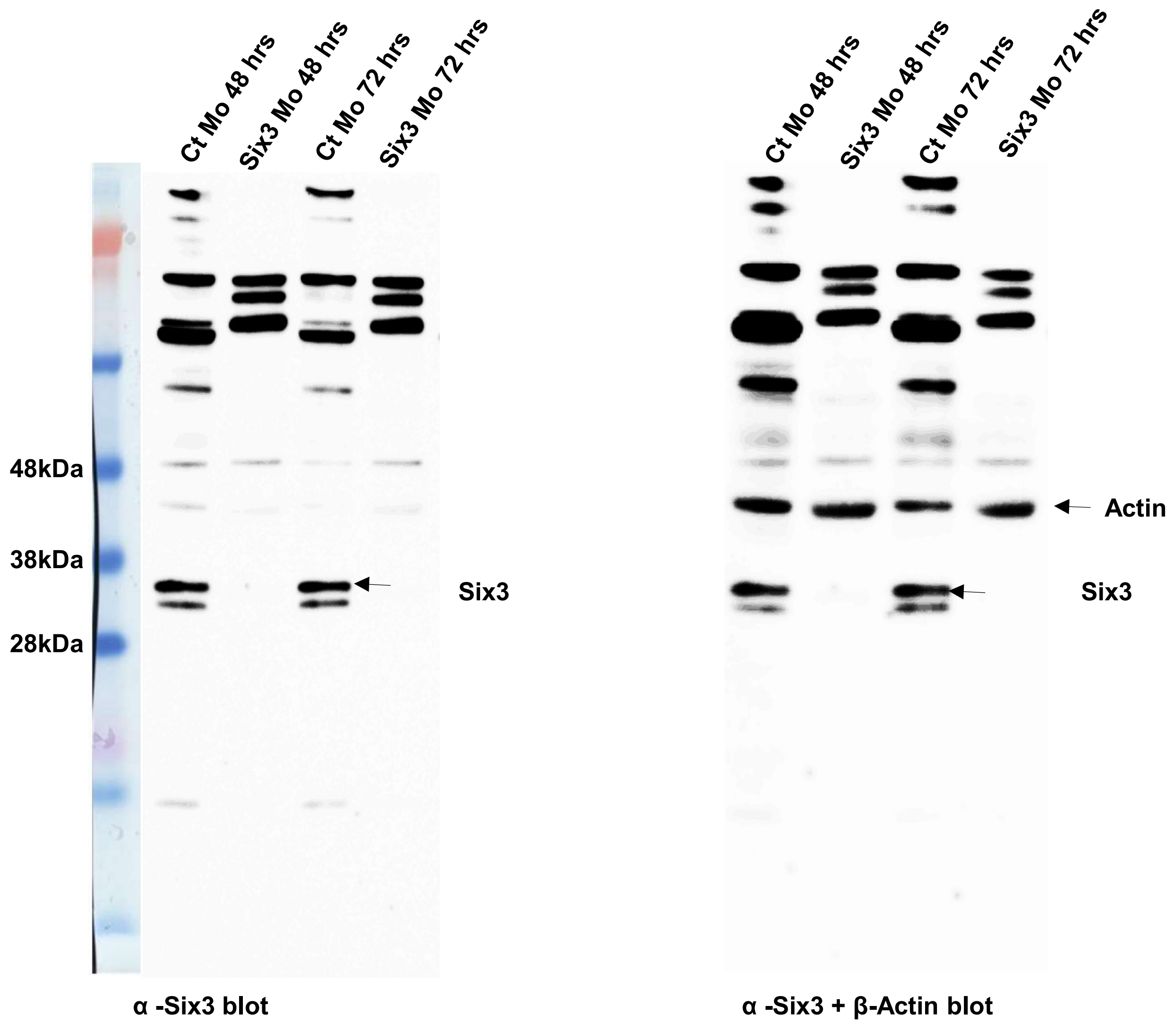

Six3 antibody detect non specific bands along with specific band (around 34kDa) which is marked with arrow.
