# Supplementary material for: Identification and functional modelling of plausibly causative cis-regulatory variants in a highly-selected cohort with X-linked intellectual disability
Source: PLoS One. 2021 Aug 13;16(8):e0256181. doi: 10.1371/journal.pone.0256181 (PMC8362966; doi:10.1371/journal.pone.0256181)
Supplement: Attachment — Submitted filename: Point by Point Reviewer Response.docx [file pone.0256181.s004.docx]

We would like to sincerely thank the reviewers for their careful reading of our manuscript and for their very helpful criticisms of the data and its presentation and their insights. We hope we have addressed all of the points raised and we feel that the paper has been enormously improved by this review process.

Reviewer #1: In this study, Bengani et al., aimed to identify variants within cis-regulatory elements on chromosome X, that could potentially be diagnostic for some undiagnosed forms of X-linked intellectual disability. While these type of studies are needed, the current paper has several substantial limitations that hinder the validity and significance of the findings (please see my “Major comments” below). The paper is also hard to read and follow, and is poorly organized (please see my “Minor comments” below).

**Major revisions:**
In the results section (line 289) where the authors describe the screening of several zebrafish line, they note the following ***“Multiple stable lines are created, expression domains are scored and only consistent differences between the reference and alternative alleles are taken as evidence of a functional effect of the mutation. Only two CRE variants in two different probands showed interpretable effects.”*** It is not clear what methods were used to evaluate/quantify these differences? The authors should add a table that shows the quantification of the signal from all lines and the cutoff (for difference) they used to unbiasedly select the two Cre variant that became the focus of this paper.

1.1

We apologise that the criteria used for selecting the CRE for further study were not explicit and the evidence not adequately documented in the supplemental information. The criteria we used to assess the enhancer function in the zebrafish were qualitative rather than quantitative. The dual colour fluorescent signals were assessed and recorded using standard microscopy in living zebrafish embryos. The scoring was based on an appreciation of consistent patterns of the anatomical distribution of the activity of the wild-type and mutant reporters in the same animal by an experienced scientist. This was one of the main reasons we were keen to use animal models in the functional analysis of CRE variants. A major advantage of using stable integration of reporters in zebrafish was that we were able to assay reporter activity in the same animals at different developmental stages. Although this approach differs from other approaches of reporter gene activity, for example in luciferase assays in cell lines, we consider it to be at least as robust and significantly more informative. The dual fluorescence reporter transgenic approach was first described in PLoS Genetics in [2013](https://journals.plos.org/plosgenetics/article?id=10.1371/journal.pgen.1003177) and then more comprehensively in [2015](https://journals.plos.org/plosgenetics/article?id=10.1371/journal.pgen.1005193). Shipra Bhatia who has pioneered this technique is a co-author on our paper and performed all the zebrafish microscopy.

The specific criteria we used for inclusion in future studies were: 1. Evidence of a consistent pattern of CRE activity in independent lines using fluorescent microscopy on live embryos. 2. Evidence of a consistent difference in CRE activity between the reference and alternative alleles in the developing brain. 3. Evidence of a significant overlap between the CRE activity and that of the orthologous zebrafish gene or genes.

We have changed the methods to better describe what has been done and we have included a table in the main text to summarise the details of the zebrafish analyses. In the supplemental information we have added a new figure which documents the phenotypic basis of the decision using multiple representative lines for each CRE variant. The figure 2 and 3 legends have been updated to include relevant links to the evidence.

In Figures 2 and 3, there need to be quantification of the imaging findings with statistical analysis. One image is not scientifically sufficient and cannot be considered as robust or valid finding.

1.2

Again we apologise for the lack of transparency with regard to replication, this was not intentional. The points regarding the zebrafish data has been addressed in the point 1.1. Quantitation of chromogenic whole mount in situ hybridisation of mouse embryos presents even greater challenges and relies even more on the qualitative assessment of the scientist performing the assay. The prime reason for this is that to prove that a signal is not present you may have to “over expose” the embryo to the chromogenic substrate. A good example of this is shown in Figure 3E; in order to be confident that there was no signal in the nasal placode the embryo was left in the substrate significantly longer leading to a spurious apparent increase in signal in the maxillary processes bilaterally. We have tried using light sheet or optical projection tomography with the RNAscope probes used in the adult brain sections in Figure 4 but the clarification of these embryos was incomplete and unsuitable for quantitative analysis. However we have now included a figure in supplementary information that shows whole-mount in situ hybridisations (WISH) on four wild-type and four Fmr1^CRE^ embryos to provide evidence of a consistent pattern – you will notice the mutant embryos all have higher background signal to ensure that we had not missed lower expression in the nasal placodes.

On reviewing the WISH data that we had recorded on the *Tenm1*^CRE^ embryos we felt that these were consistent but inadequately documented to convince either a reviewer or reader. We have therefore removed these images from Figure 2 and removed from the text any reference to the altered developmental brain expression in this line.

Western blot analysis includes only one sample per genotype per brain region. This can’t be considered a scientifically valid results. If the authors did analyze more samples are just presenting representative figures, then they should provide the quantification and statistical analysis data.

1.3

We agree with this point regarding western blot analysis and apologise for the confusion. We have moved the FMRP western blot that shows the four replicates of P25 hippocampal slices from the wild type and *Fmr1*^CRE^ mutant mice from the Supplementary file to Fig 4. This is now more logically sited next to the graph showing the quantitation. The details of the statistical analyses have been added to the main text methods and the figure legend has been updated. We agree this significantly improves the flow of the paper. We have removed the P14 FMRP western blots. Unfortunately we only have duplicate blots at this stage and using a 95% upper and lower confidence interval on the FMRP:Actin intensity ratio there is tentative evidence of higher levels of FMRP in the hindbrain.

Number of animals that were tested on the behavioral paradigm and other experiments are not listed. The age of sex of animals for most experiments is not listed. There is no description for any statistical analysis.

1.4

We apologise for the lack of clarity in Figure 4. All experiments used only male animals at post-natal day 25 (P25). We have added the numbers used in each component to the figure and we have moved the details of the statistical analysis are in the main text.

If there are no changes in Fmr1 levels (at least as tested by qPCR although also protein levels quantification should be done in at least n=4 animal per mouse line), then how can the authors explain the effect on protein synthesis?

1.5

We agree this is one of the most interesting findings in the paper. There is actually *increase* in FMRP protein levels when comparing hippocampal slices from *Fmr1*^CRE^ and wildtype P25 male mice. This was unexpected for two reasons: 1. There is no obvious increase in *Fmr1* transcript levels. 2. There is increased bulk protein translation (most commonly documented in Fmr1-null male animals). Our favoured explanation is that earlier developmental dysregulation of Fmr1 levels has altered the hippocampal cell state. This cell state change may be a consequence of the reduced levels of *Fmr1* transcript we observed in the embryonic midbrain by WISH (see pont 1.2). We acknowledge that this requires more work to provide an adequate explanation but this is not within the scope of this paper.

In the methods’ section (line 151), the authors note that one of the approaches they took to evaluate the variants, was based on “their association to target genes known to be responsible for X-linked ID”. It is not clear how was this “association” determined or based on?

1.6

The association between the CRE and its target gene was the subject of a previous paper we published in Nature Communications: Naville et al. Long-range evolutionary constraints reveal cis-regulatory interactions on the human X chromosome. Nat Commun. 2015 Apr 24;6:6904. doi: 10.1038/ncomms7904. PMID: 25908307; PMCID: PMC4423230. We have added a brief summary of this strategy to the main text

The authors refer to “supplementary note” but there is no specification as to where this information would be found. Please be specific when referring to supplementary data.

1.7

We apologise for this omission. We have added a table of contents and page numbers to the supplementary information and added specific references to the main text.

In the “author summary”, the authors say: “Here we have attempted to identify all CRE on the X chromosome” while in other sections of the paper, it seems that these CREs have been identified elsewhere and the author used the coordinated of these previously identified/predicted CRE to generate primers for sequencing. This kind of statements need to be more clear and consistent across the paper. Also, what do you mean by “rational” approach? Also here, please be specific and clear with the choice of words.

1.8

We apologise for this error. This relates to the problem identified in point 1.6. We have changed this to: *“These baits were designed to capture two non-overlapping sets of target sequences from the human X chromosome (chrX); all chrX coding exons and all chrX CRE. The set of chrX CRE, accounting for 4.4% of chrX genomic sequence, had been defined in a previous study [15]. This study also showed that the maintenance of linkage between a CRE and neighbouring genes throughout evolution was an accurate way to identify the target gene(s). Target genes assigned using this conserved synteny approach allowed a maximum CRE to gene distance of 1.5 Mb [15]. Approximately a third of chrX CRE could be assigned to a single gene with the remainder having >1 equally plausible target. 389/812 protein coding genes on the X chromosome could be assigned to at least one CRE.”*

As mentioned in point 1.6 we will ensure that the strategy is briefly explained in the main text.

Minor comments:

Using the term “undiagnosed neurodevelopmental disease” is confusing and perhaps contradicting itself, when knowing that we are in fact talking about neurodevelopmental disease [please use “disorder” instead because ID is not a disease]. I would recommend saying “Neurodevelopmental disorders with unidentified etiology”

1.9

We completely agree that this phrase is ambiguous and potentially misleading. We have changed the first sentence of the abstract to read: *“Identifying causative variants in cis-regulatory elements (CRE) in neurodevelopmental disorders has proven challenging.*” We have changed the only other instance of this in the main text to the phrase suggested by the reviewer.

Please be more accurate and clear with the language. For examples, what do you mean by targeted sequencing (in the abstract)? unique vs. common variants…etc.

1.10

We have changed the sentence in the abstract to clarify the meaning of targeted sequencing in this context. We have been through the entire text to ensure that such vague or ambiguous terms are defined the first time they are used.

In the methods section, the authors note that the “The patients had previously been tested negative by routine diagnostic approaches (i.e., CGH microarray analysis at 500 kb resolution, fragile X [MIM #300624], methylation status of Prader Willi [MIM #176270]/Angelman syndrome [MIM #105830]). In addition, coding variants on the X chromosome likely to lead to disease have not been found within a previous study [15]”. Yet they don’t clarify if these 48 patients were part of that study (reference 15). While this is clarified in the Results section, I still think that it should as well be clarified in the methods section as it is a very important piece of information.

1.11

We apologise for this omission. We have clarified the results to confirm that all 48 of these families were part the previous study

The following statement in the Results section (line 252) “The prior probability that any causative CRE variant would be on X chromosome also significantly reduced the genomic search space of interrogation” is very vague. Please clarify.

1.12

We have re-phrased this term to read: “We reasoned that these families are likely to be enriched for highly penetrant causative regulatory mutations. In addition, the high prior probability that any causative variant in these families would be located on the X chromosome significantly reduced the genomic space for interrogation. A summary of the overall study design is presented in Fig 1A.”

Results (line 306) please explain the rational of using morpholinos (you got to assume that there are non-zebrafish experts in the field).

1.13

We have added the following text to explain the rationale for using the mopholinos: “*To determine if SIX3-mediated repression may be responsible for the altered enhancer activity in the variant TENM1^CRE^ we chose to use morpholino-induced knock-down of endogenous six3 in TENM1^WT^/TENM1^CRE^ transgenic embryos. The phenotypic effect of the morpholinos targeting zebrafish six3 was assessed by 1-cell embryo injections. The amount of morpholino was titrated to the point where there was no morphological anomaly see at 24 hours. When this concentration of morpholino was injected into TENM1^WT^/TENM1^CRE^ transgenic embryos there was rescue of the activity of mutant CRE in the midbrain and hindbrain with no effect on the wild-type reporter (Fig 2F and 2G). This supports the hypothesis that the CRE variant had created a repressive SIX3 binding site as the mechanism for the transcriptional effect in zebrafish embryos.*”

Figure 2 and 3 mixes mice and zebrafish data and the title of Figure 3 is explicitly about mice (“FMR1 associated regulatory variant alters the expression of Fmr1 in mouse embryonic development.”) yet the figure includes zebrafish data. Please re-arrange the figure to make a smoother flow.

1.14

We have removed the mouse embryo data from Figure 2 and we have renamed this “***Figure 2: TENM1^CRE^ alters enhancer function in the zebrafish brain by creating a repressive Six3 binding site****.*”. Figure 3 has been renamed: “***Figure 3: FMR1^CRE^ alters enhancer function in the zebrafish brain****.*”

The order of the figures as they appear in the text is confusing (for example Fig 4G appears before Fig 4a or 4B). Please make sure the number and alphabetic orders are listed in an adequate order in the text.

1.15

We agree that this is a problem and to improve the flow of the data we have re-organised Figures 3 & 4 to match the order of reference in the text. The new Figure 4 has only mouse data on the levels of *Fmr1* transcripts (including the mouse embryo data that was in Figure 3) and FMRP protein. Figure 5 now has only the olfactory test data. Figure 6 has the long term depression electrophysiology, the audiogenic seizure predisposition study and the bulk protein synthesis assay. The legends have been reconciled with the new figures.

**Reviewer #2: Summary**

In this study, the authors sought to identify causative non-coding variants in 48 males with X-linked intellectual disability, who had been previously shown to have no mutations in coding regions. It is a clever approach, in that the authors focus on a specific group of patients with only one chromosome responsible for their intellectual disability in order to narrow down the search for potential non-coding variants. The authors use both zebrafish and mouse models to explore potential non-coding variants. The presented data supporting the causative role for the FMR1-CRE variant is convincing, making this manuscript a valuable example of deciphering the role of non-coding mutations. However, the manuscript is somewhat sloppy, such as inconsistent and seemingly random text bolding, and needs a careful cleanup before publication (see minor comments, and I may have missed some). Additionally, there is a lack of details and logic to the writing. Specifically, more details should be included to explain reasoning for approaches taken and conclusions drawn (see major comments).


Major Comments

-Authors talk about rare variants and differentiate between rare and rare but are too common to be likely disease-associated. What does this even mean? Is there a quantification for this? I see that there is a reference for it (line 396), but it should be explicitly explained. Also, if 3/6 of the six variants are in this category, why did the filter down to only 6 variants (see next point)?

2.1

This is a very important criticism; addressing this will significantly clarify the flow of the information and message we hope to convey. A factor of major clinical significance that, on re-reading, is not clear in the text is the length of time required to complete the necessarily sequential experimental processes we have described in the manuscript. We performed the targeted sequence analysis in 2015 and at that point the only publicly available sources of whole genome data we had available was from the 1000 genomes and UK10K studies. This allowed us to screen out common non-coding variants (allele frequencies > 0.01) but the scale of this data did not allow us to differentiate rare (AF 0.01-0.0001) from ultrarare (<0.0001 and unique) variants. The 6 variants at that point seems plausibly disease associated as they were, at that time, unique.

Importantly our reinterpretation of the variants mentioned above only occurred after the release of the gnomAD 2.1 WGS data in 2018 in conjunction with the paper by Whiffen et al. describing their approach to calculating the maximum plausible allele frequencies for any highly penetrant genetic disease (PMID: 28518168). By this time, we had already completed the zebrafish dual-colour fluorescent reporter analysis and made the Fmr1^CRE^ and Tenm1^CRE^ mouse lines and begun the behavioural and expression analysis. We have made the following changes to try to improve coherence and comprehensibility:

A. We have added a diagrammatic summary of the experimental logic and order to Figure 1A. To make room for this we have moved the details of the filtering (previously Figure 1A) to the supplementary data as Supplemental Figure 1.
B. We have changed the order of the methods and results to reflect the diagram in Figure 1A

-Given that the vast majority of the tested 31 variants did not segregate as expected, would it not make sense to have screened more comprehensively. Perhaps starting with the 9,577 variants after filtering for common variants? Or starting after one of the subsequent filters (such as with the 628 rare variants)? This is likely beyond the scope of the study, but it seems important to know all potentially causative variants for each of the 48 patients. The filtering steps are reasonable, but ending up with only six potential variants and one confirmed indicates that a lot of information was missed and that the pipeline could be improved. Please discuss how to go about identifying causative variants for the other 47 patients and at what points the pipeline most likely needs modification. I would think that screening for segregation should happen on many more variants, but there may be limitations in cost?

2.2

There is no doubt that the filtering we used was designed to identify variants with the highest prior probability of interpretable effects. There is also no doubt that we may have missed causative variants, particularly those that affect genes that are not currently know to cause XLID. The reviewer is correct that we had to balance the scale of the investigation to the available resources. We take the point that it is important to discuss how to investigate the other 47 families in whom we have failed to identify a cause. We have therefore added the following section to the discussion: “*Our experimental design can be justified on pragmatic grounds. However, we do recognize at least two significant problems with this filtering strategy. First, the CNE variant could induce expression in cells types where the target gene is normally silenced, which is likely to result in a phenotype completely distinct from that associated with intragenic mutations. Secondly, if intragenic mutations in the target gene result in early embryonic lethality this gene would not be identified as a cause of XLID. However, mutations in a CRE controlling only neural expression of an essential gene could cause XLID but would be missed in our analysis which is focussed on known XLID genes.*”

-The authors refer to the TENM1-CRE variant as “likely neutral”, even in the abstract, but it’s not clear why this is? Was it because of the allele frequency (lines 401/402)? - is the TENM1-CRE variant one of those that is not actually rare? Or is it because they do not have behavioral defects, as is mentioned in the abstract (“However, . . . defects”). There is no requirement for mouse behavior to mimic human behavior, so I don’t think the annotation of this variant as neutral would be due to the behavior findings (especially since so few assays were tested). Please clarify the interpretation and reasoning for why the authors consider this variant neutral (around lines 401/402). If it is due to allele frequency, then why not go back to the set of 628 variants (or 9,577) and be more stringent on allele frequency? There is more explanation on 437-441, but I do not understand why the TENM1-CRE was even included in the final six variants if the allele frequency was too high (since allele frequency based gnomAD was part of the filtering of the 9,577 variants down to 31). I absolutely agree with the interpretation the altered gene expression should not be considered evidence for disease association, but I am confused and need more information. Also, on line 419 the authors indicate that the olfactory test was plausible for the TENM1-CRE, but this does not make sense based on the expression changes. Please also further explain the choice of behavior assay.

2.3

We recognise and apologise that this was not clear, as it is a key point for us. We fully agree that the behavioural assay results cannot be taken as strong evidence against disease-association. The assessment that *TENM1*^CRE^ is unlikely to be disease associated is based entirely on the fact that its frequency in the background population exceeds the calculated likely plausible allele frequency for a XLID-causing variant. We hope that the change in the order of the results outlined in point 2.1 will clarify this.

-While morpholinos are notoriously suspect and there were better experiments the authors could have done instead, the question being asked with the morpholino experiment is simplistic enough that the interpretation appears valid. This experiment also seems sort of unrelated to the rest of the story that focuses on FMR1, so most conclusions are not based exclusively on a morpholino experiment. However, it is worth briefly noting the limitations of morpholinos, so that it is clear to the reader that the authors have considered this controversial approach.

2.4

We hope that the change in the order of the results section mentioned in point 2.1 will help address this problem. This experiment was done because, at the time, we still considered this variant to be plausibly causative. It was only with the release of the gnomAD WGS derived data that it became clear that this was unlikely. We feel it is important to keep the morpholino data in the result section as it emphasises that even if you can explain a change in enhancer function at a molecular level it does not mean that this is causative.

- My biggest concern with the manuscript is that the zebrafish data lacks numbers of animals screened and statistical information regarding the expression findings. Showing a single example is not sufficient. There is too much variability in zebrafish development and gene expression to rely on a single image. The internal GFP control is essential, but it does not replace the need to quantify the findings. I see there are some numbers in the supplementary information, but it’s not clear if this is the number of larvae examined or the number of unique transgenic lines that produced larvae (and then how many larvae were looked at from each?). I may have missed this information, but I can’t seem to find it and it needs to be more prominent if it is included.

2.5

We apologise for this omission which was also a major concern for Reviewer 1 (see points 1.1 and 1.2 above). We have added a table to the main text detailing the results of the zebrafish screen and more importantly add a figure to the supplementary information that documents the evidence.

-To what extent are the sequences of the FMR-CRE and TENM1-CRE regions conserved in zebrafish? What about the specific human mutations? If they are conserved, why make mice instead of making zebrafish? The choice of experiments is fine, but the explanation and reasoning of going back and forth between the species could be further detailed. Is the SIX3 binding site also in the native zebrafish CRE if the corresponding variant was made in zebrafish?

2.6

This is a very interesting point that we have not adequately addressed in the paper (or indeed the literature). We have added a figure to the supplementary data (S42 Fig) showing the mammalian, chicken, xenopus and zebrafish alignments of each of the six CRE that had variants identified in Figure 1. In spite of very high vertebrate conservation scores, there is no detectable conservation of the primary sequence of the CRE in zebrafish for any of these CRE. This was the reason that we had to model the variants in mice. We have added the following text to the discussion to highlight the unusual phenomenon of zebrafish reporting enhancer activities that seem to overlap with their endogenous gene activity but without any obvious orthologous CRE being present in their genome: “*It is remarkable that, in absence of obvious homology with human CRE sequences (S42 Fig), the developing zebrafish brain appears to report the same transcription factors to control site and stage specific gene activation. This argues for a subtler shape-based interaction of DNA with transcription factors that we are, as yet, unable to understand. Defining the grammar of this structural effect will significantly aid our interpretation of variants in the non-coding genome*.”

-Why were seizures tested? Are there seizures in the patients? What was the significance of the choices of behaviors to test?

2.2

Audiogenic seizures are a very highly penetrant feature in *Fmr1*-null mice. The fact that that the *Fmr1*^CRE^ mice did not have increased audiogenic seizures suggests that AGS are a direct consequence of the lack of FMRP in the adult brain rather than during embryogenesis. To clarify this we have added the following text to the results section: “*A predisposition to audiogenic seizures is also a consistent phenotype in Fmr1-null mice but Fmr1^CRE^ male mice showed no increase in such seizures (Fig 6C).*”

Minor Comments

Figure citations: not consistent in whether they are bolded or the use of a period after Fig. Please go through and make all of these references consistent. Do the same for tables and all referenced supplementary information, such as the supplementary note. There are also issues with bolding in figure legends.

We have standardized all of the in-text references to: **Fig X, Table X, SX Fig, SX Note, SX Table.** We have reviewed the figure legends for consistency

Line 42: variants should be variant

Changed

Line 43: comma after subsequently
Sentence altered

Line 44: change “that was” to “, which is”
Sentence altered

Line 47: it’s not immediately clear that the authors are referring to the TENM1-CRE variant here. There needs to be a better connection here, so that the reader does not think that the “neutral” variant is FMR1-CRE (from prior sentence). Please rewrite for clarity.
Sentence altered

Line 153: S4 table is listed before S2 and S3 tables. They should be referenced in order.
Tables are now ordered appropriately

Line 165: “wild type” should be “wild-type” because it’s an adjective for versions.
All examples of wild type have been changed to wild-type

Line 167: it would be helpful to the reader to know the approximate sizes of the CREs and the way the constructs are set up. I would add a diagram to the Figures 2/3 to lay out the construct. I am aware the primers are given, but it would be a lot easier for the reader if additional relevant information was also given to understand the experiment better.
Changed

Line 168: It’s a little tricky that the S2 Table is inside the Supplementary Information document. Should it be a separate table or is there a more specific way it can be referenced so that it is easy to find? Not sure about the requirements for this journal, but it wasn’t immediately obvious where it was because the S1 and S4 tables are separate documents.
The supplementary information document has been completely revised and now has a table of contents. **S2 Table** in the original submission is now **S3 Table** in the revised version

Lines 168, 169, 180, 196, and 274: If you are saying something is “listed/shown in” you should not put the table you are referring to in parentheses. Could keep in parentheses if you reword the sentence to not say “in”.
All corrected

Lines 173 and 176: Use prime symbols instead of apostrophes
All corrected

Line 184: “A double” not “double”.
All corrected

Line 188: sentence is awkward and could be improved (“is used” and “using”)
Changed to: The guide RNA is generated from this PCR template using T7 RNA polymerase (NEB), and purified with RNeasy mini kit (Qiagen) purification columns.

Line 189: NEB
Corrected

Line 190: “contains” should probably be “contained”. There is overall some issues with tense-switching in this paragraph, and it would be better to keep a consistent tense.
Sentence altered

Lines 202/203: Inappropriate comma usage
Sentence altered

Line 204: space between 1 and cm
Sentence altered

Line 249: add comma after unrelated males
Sentence altered

Line 251: change “cohort are” to “cohort is” or change phrasing in some other way
Sentence altered

Line 292: “There” is not the appropriate word choice. Change the wording of this sentence.
Sentence altered

Line 378: add comma after “sequencing”
Comma added

Line 383: missing space before reference [39]
Sentence altered

Line 388: “limiting” should be “limit”
Sentence altered

Line 389: add comma after “genes”
Comma added

Line 396: add “an” before “established”
Sentence altered

Line 400: should be a colon, not a semi-colon
Sentence altered

Line 435: add comma after “that”
Comma added

Line 437: replace “class” with “classify”
Sentence altered

Figures 2 and 3: labeling of specific parts of the brain should be consistent (2D) and clear (3C, 2D, 2E)
Changed

Figure 3: bounding boxes/image panels should all be the same size and in line with each other
Changed

Figure 4: (A) Straighten by rotating western blot (D) super hard to discern anything but that is probably due to the low image quality mentioned earlier
Figure completely revised

Figure 4 legend: This whole text is sloppy, but particularly see below lines:
Legend completely revised

517: Green and Blue should be lowercase
Changed

518: Space after period, remove s from transcripts, replace are with is, relace spots with a spot (or dot)
Changed

520: space between male and (purple) and with next line
Changed

521: fix space to be after period, not before
Changed

524: hyphen in wild-type, littermates one word

Changed
